# Evidence of a true pharyngeal tonsil in birds: a novel lymphoid organ in *Dromaius novaehollandiae* and *Struthio camelus* (Palaeognathae)

**DOI:** 10.1186/1742-9994-9-21

**Published:** 2012-08-21

**Authors:** Martina R Crole, John T Soley

**Affiliations:** 1 Department of Anatomy and Physiology, Faculty of Veterinary Science, University of Pretoria, Private Bag X04, Onderstepoort, 0110, South Africa

**Keywords:** *Dromaius novaehollandiae*, *Struthio camelus*, Pharyngeal tonsil, GALT, *Lymphonoduli pharyngeales*

## Abstract

**Background:**

Tonsils are secondary lymphoid organs located in the naso- and oropharynx of most mammalian species. Most tonsils are characterised by crypts surrounded by dense lymphoid tissue. However, tonsils without crypts have also been recognised. Gut-associated lymphoid tissue (GALT), although not well-organised and lacking tonsillar crypts, is abundant in the avian oropharynx and has been referred to as the “pharyngeal tonsil”. In this context the pharyngeal folds present in the oropharynx of ratites have erroneously been named the pharyngeal tonsils. This study distinguishes between the different types and arrangements of lymphoid tissue in the pharyngeal region of *D. novaehollandiae* and *S. camelus* and demonstrates that both species possess a true pharyngeal tonsil which fits the classical definition of tonsils in mammals.

**Results:**

The pharyngeal tonsil (*Tonsilla pharyngea*) of *D. novaehollandiae* was located on the dorsal free surface of the pharyngeal folds and covered by a small caudo-lateral extension of the folds whereas in *S. camelus* the tonsil was similarly located on the dorsal surface of the pharyngeal folds but was positioned retropharyngeally and encapsulated by loose connective tissue. The pharyngeal tonsil in both species was composed of lymph nodules, inter-nodular lymphoid tissue, mucus glands, crypts and intervening connective tissue septa. In *S. camelus* a shallow tonsillar sinus was present. Aggregated lymph nodules and inter-nodular lymphoid tissue was associated with the mucus glands on the ventral surface of the pharyngeal folds in both species and represented the *Lymphonoduli pharyngeales*. Similar lymphoid tissue, but more densely packed and situated directly below the epithelium, was present on the dorsal, free surface of the pharyngeal folds and represented a small, non-follicular tonsil.

**Conclusions:**

The follicular pharyngeal tonsils in *D. novaehollandiae* and *S. camelus* are distinct from the pharyngeal folds in these species and perfectly fit the classical mammalian definition of pharyngeal tonsils. The presence of a true pharyngeal tonsil differentiates these two ratite species from other known avian species where similar structures have not been described. The pharyngeal tonsils in these ratites may pose a suitable and easily accessible site for immune response surveillance as indicated by swelling and inflammation of the tonsillar tissue and pharyngeal folds. This would be facilitated by the fact that the heads of these commercially slaughtered ratites are discarded, thus sampling at these sites would not result in financial losses.

## Background

Tonsils (*Tonsilla pharyngea*) are secondary lymphoid organs located in the naso- and oropharynx and are found in most mammalian species, except rodents [1]. Tonsils form part of the well-organised mucosa-associated lymphoid tissue (MALT) [2]. This tissue occurs at strategic sites in the body and is involved in sampling antigens from mucosal surfaces and the induction of immunity at these sites [2]. GALT (gut-associated lymphoid tissue) is a sub-division of MALT [2] and is found in the digestive tract. In mammals, tonsils are present in the pharynx at distinct anatomical sites [2] forming what is referred to as Waldeyer's ring which guards the entrance of the nasal, oral and auditory passages into the pharynx [2]. Tonsils forming this ring are the tonsil of the soft palate and the lingual, palatine, paraepiglottic, tubal and pharyngeal tonsils [3]. Tonsils therefore constitute the initial defence of the body to inhaled and ingested harmful organisms [3].

Unlike the situation in mammals, there are no reports of a tonsillar ring in birds. However, lymphoid tissue is abundant in the avian oropharynx [4], and is found concentrated in the pharyngeal region [5-7]. In birds this lymphoid tissue has been referred to as the “pharyngeal tonsil” [4,8,9] and, together with various concentrations of lymphoid tissue found throughout the avian digestive tract, including esophageal, pyloric, and caecal tonsils, has been classified as GALT [9]. The term “tonsil”, when applied to the pharyngeal tonsil in birds, has been used unadvisedly when compared to the definition of tonsils in mammals. In the fowl, for example, the lymphoid tissue around the choanal and infundibular clefts, although not well-organised and lacking tonsillar crypts, has traditionally been termed the pharyngeal tonsil [9]. In contrast, the mammalian tonsils are described as complex structures characterised by crypts surrounded by dense lymphoid tissue [10], although tonsils without crypts have also been described [1].

In ratite species, the roof of the oropharynx caudal to the choana is characterised by the presence of large, paired, U-shaped structures referred to as pharyngeal folds (*Plicae pharyngis*) and these structures have been described in *D. novaehollandiae*[11] and in *S. camelus*[12-14]. Pharyngeal folds in ratite species have previously been referred to as ‘tonsils’ [15]. In *D. novaehollandiae*, the large amount of lymphoid tissue present in the pharyngeal folds led to naming the associated glandular field the *Gl. tonsilla pharyngea*[16], whereas in *S. camelus* the abundance of lymphoid tissue in the pharyngeal folds prompted the conclusion that these structures represent immunologically active tonsils [14]. Although they contain significant concentrations of lymphoid tissue, the pharyngeal folds in ratites do not represent true tonsils as defined in mammals [3]. In *S. camelus*, Tadjalli et al. [13] identified a separate structure associated with the pharyngeal fold as a pharyngeal tonsil. Although the structure was identified as such, no histological evidence supporting its tonsillar nature was provided. Thus evidence for the presence of true tonsils in the avian oropharynx remains elusive.

This morphological study presents the first detailed description of distinct anatomical structures in the oropharynx of *D. novaehollandiae* and *S. camelus* that represent true pharyngeal tonsils (*Tonsilla pharyngea*) in avian species. A distinction is made between the lymphoid tissue present throughout the ventral surface of the pharyngeal folds (*Lymphonoduli pharyngeales*) and specific aggregations of GALT associated with the folds that fit the classical description of pharyngeal tonsils (both follicular and non-follicular) as described for mammals. This lymphoid tissue-rich region, which is the first site of contact for ingested antigens, warrants further investigation. The existence of typical tonsils in these two commercially important birds may open new avenues for the development of oral vaccines in ratite species. Furthermore, evaluating these mucosal surfaces in *D. novaehollandiae* and *S. camelus*, in both living birds and birds slaughtered at commercial abattoirs, may form an important aspect of immunopathology and immune response surveillance, particularly as the mucosal immune system can reportedly act independently of the systemic immune system [17].

## Results

### Gross morphology

**Figure 1:**
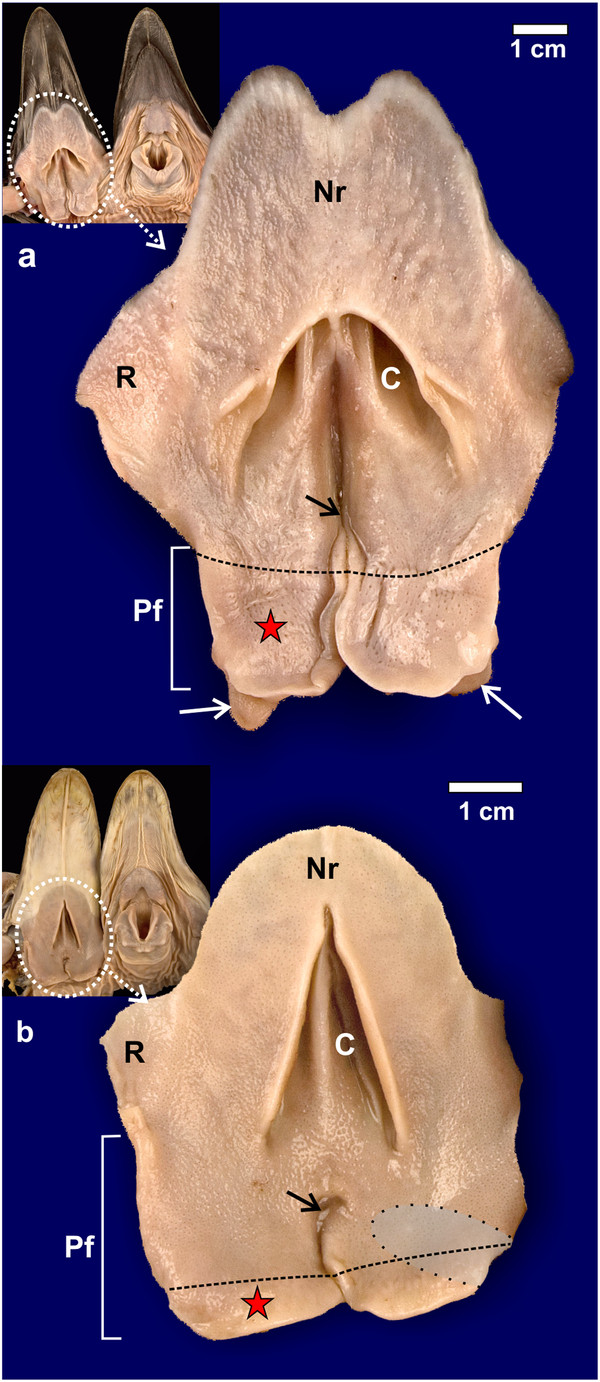
**Gross morphological features of the non-pigmented roof (Nr) of the oropharynx.a**: *D. novaehollandiae*. **b**: *S. camelus*. Choana (*C*), rictus (*R*) and paired pharyngeal folds (*Pf*). The black dotted line on the pharyngeal folds denotes the border between the free (*red star*) and attached regions of the folds. The infundibular cleft (*black arrow*) is more obvious in *S. camelus* than in *D. novaehollandiae*. Caudo-lateral extensions of the pharyngeal folds (*white arrows*) are obvious in *D. novaehollandiae.* The retropharyngeal position of the underlying pharyngeal tonsil in *S. camelus* is indicated (*grey shaded area*). Insets show the openly displayed oropharynx of *D. novaehollandiae* and *S. camelus*.

**Figure 2:**
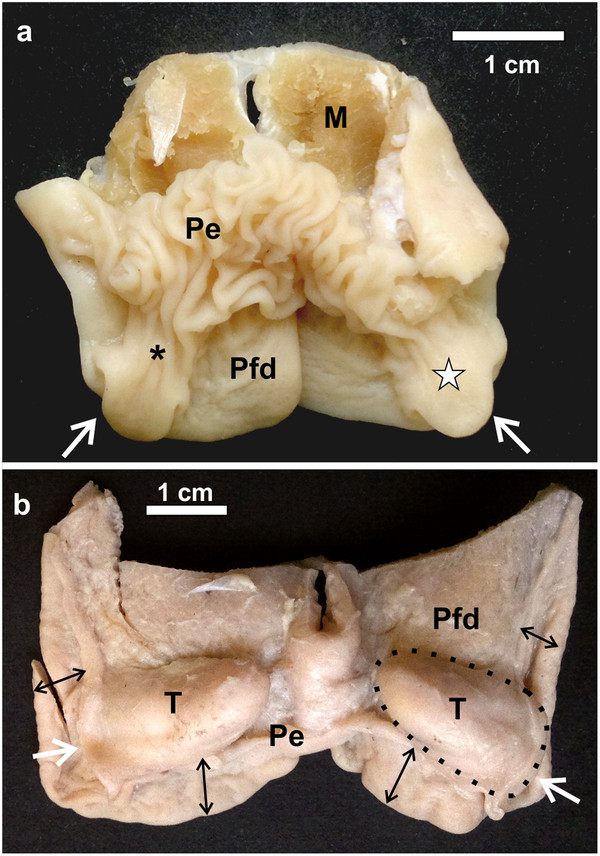
**Dorsal view of the pharyngeal folds and associated follicular tonsils. a**: *D. novaehollandiae*. The pharyngeal tonsil (*white star*) is situated entirely within the recess formed between the dorsal, free surface of the pharyngeal folds (*Pfd*) and the proximal esophagus (*Pe*) which is continuous with the base of the caudo-lateral extension (*). The opening of the tonsil to the oropharynx is indicated (*white arrows*). Muscle tissue (*M*). **b**: *S. camelus*. The opening of the pharyngeal tonsils to the oropharynx is indicated (*white arrows*). The proximal esophagus has been removed to expose the retropharyngeally positioned pharyngeal tonsils (*T*) (*black dotted outline*) as well as the dorsal aspect of the short free part (*black double-headed arrows*) of the pharyngeal folds (*Pfd*).

**Figure 3:**
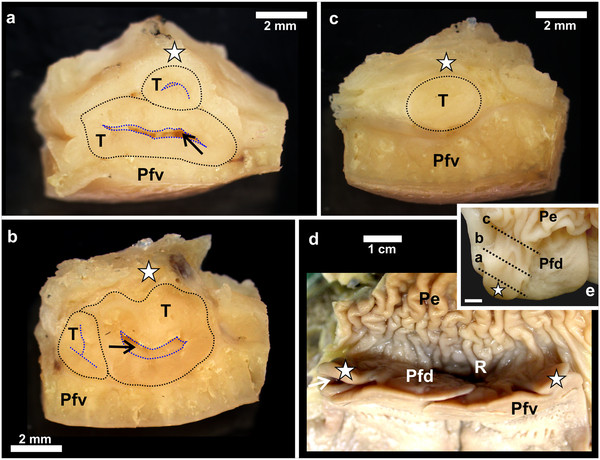
**Gross morphological features of the follicular pharyngeal tonsil of *D. novaehollandiae. *a**-**c**: The pharyngeal tonsil (*T*) (*black dotted outline*) is shown sectioned through the planes a-c as indicated in Figure 3e. Note the positioning of the tonsil between the caudo-lateral extension (*white star*) and the ventral surface of the pharyngeal fold (*Pfv*). The tonsil displays a central crypt (*black arrow*) from which numerous smaller crypts branch (*blue dotted outline*). **d**: The recess (*R*) enclosed by the dorsal surface of the pharyngeal folds (*Pfd*) with the attached caudo-lateral extension (*white star*) and the proximal esophagus (*Pe*). Ventral surface of the pharyngeal folds (*Pfv*) and opening of the tonsil into the oropharynx (*white arrow*). **e**: Dorsal surface of the pharyngeal fold (*Pfd*) with the caudo-lateral extension (*white star*) and attached proximal esophagus (*Pe*). Black dotted lines (*a-c*) indicate the plane of sectioning corresponding to Figures 1a-c. Bar = 4 mm.

**Figure 4:**
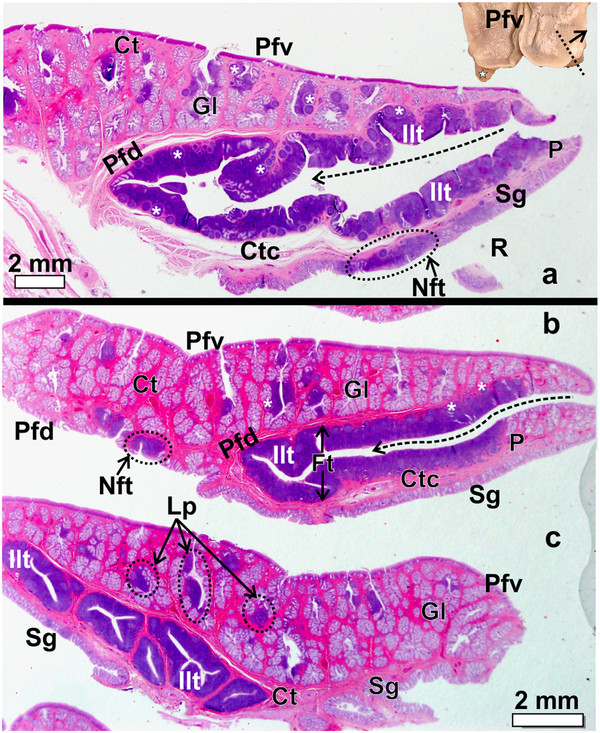
**The pharyngeal fold and caudo-lateral extension with enclosed pharyngeal tonsil in *D. novaehollandiae. *a**-**c**: Progressively more lateral longitudinal sections as indicated by the dotted line in the inset. Note the numerous simple, branched, tubular mucus-secreting glands (*Gl*) and associated lymph nodules (*) and inter-nodular lymphoid tissue surrounded by connective tissue (*Ct*) present on the ventral surface of the pharyngeal fold (*Pfv*). The follicular pharyngeal tonsil (*Ft*) is formed by a large number of lymph nodules and inter-nodular (*Ilt*) lymphoid tissue enclosed between the dorsal surface of the pharyngeal fold (*Pfd*) and the ventral surface of the extension (*P*). The extension is supported by a core of connective tissue (*Ctc*). The dorsal surface of the extension contains simple, tubular mucus-secreting glands (*Sg*). Note the long crypt (*dotted arrow*) which branches. Non-follicular tonsil (*Nft*). The recess between the dorsal surface of the pharyngeal fold and proximal esophagus (not on figure) is indicated (*R*). **c**: Longitudinal section near the lateral edge of the extension showing the well-encapsulated lymphoid tissue surrounded by connective tissue and the branching of the tonsillar crypts. *Lymphonoduli pharyngeales* (*Lp*) are associated with the glands but not situated directly below the epithelium. Inset: Caudo-lateral extension (*star*).

**Figure 5:**
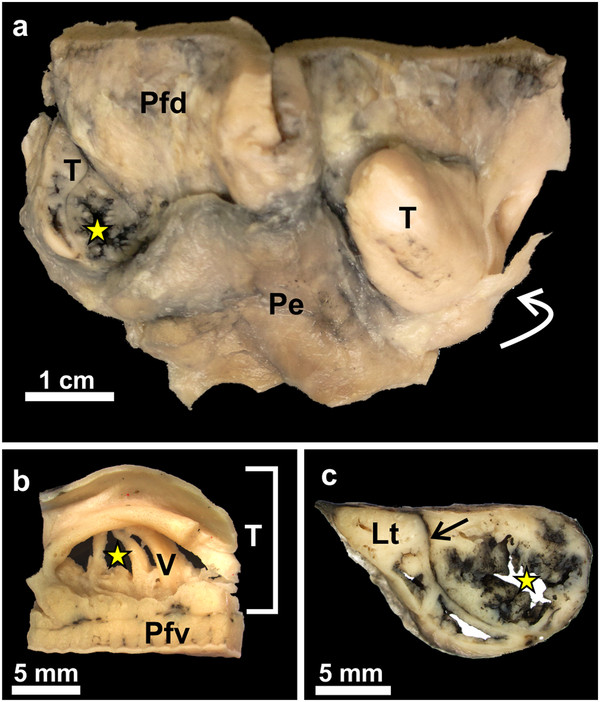
**Gross morphological features of the follicular pharyngeal tonsil of *S. camelus. *a**: The pharyngeal tonsil on the left has been injected with Indian ink and sectioned to show the tonsillar crypts (*yellow star*). Dorsal surface of the pharyngeal fold (*Pfd*), adventitia of the proximal esophagus (*Pe*) and opening of the tonsil to the oropharynx (*curved white arrow*). **b**: View of the opening of the pharyngeal tonsil (*T*) as seen from the oropharynx. The pharyngeal fold (*Pfv*) forms its ventral border. Note the vertical vanes (*V*) and the intervening tonsillar crypts (*yellow star*) opening into a shallow tonsillar sinus. **c**: Cross-section of the tonsil injected with Indian ink. The ink has stained the lining of the crypts (*yellow star*) as well as the intervening connective tissue (*arrow*). The lymphoid tissue (*Lt*) appears a cream colour.

**Figure 6:**
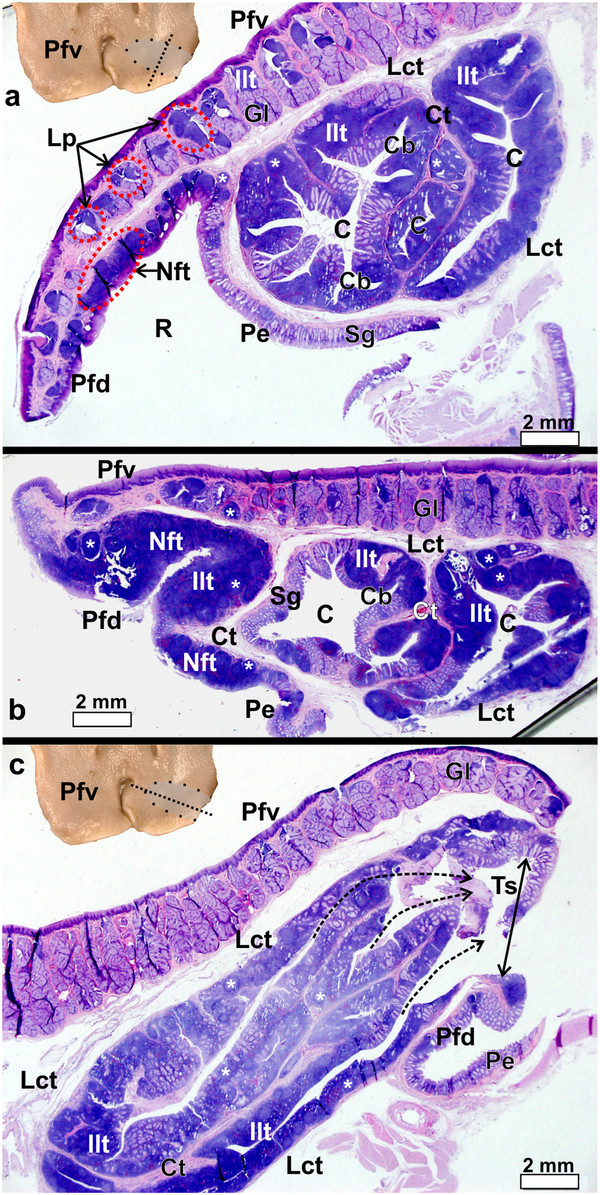
**Pharyngeal fold and pharyngeal tonsil of *S. camelus. *a**-**b**: Transverse sections as indicated by the dotted line in the inset. Note the numerous simple, branched, tubular mucus-secreting glands (*Gl*) and associated lymphoid tissue surrounded by connective tissue (*Ct*) present on the ventral surface of the pharyngeal fold (*Pfv*). *Lymphnoduli pharyngeales* (*Lp*) are associated with the ventral surface of the folds. The dorsal surface of the pharyngeal fold (*Pfd*) displays substantial amounts of both lymph nodules (*) and inter-nodular (*Ilt*) lymphoid tissue which in places forms a non-follicular pharyngeal tonsil (*Nft*). The follicular pharyngeal tonsil is formed by a concentration of lymph nodules and inter-nodular lymphoid tissue positioned between the ventral surface of the pharyngeal fold and the proximal esophagus (*Pe*) and is encapsulated by loose connective tissue (*Lct*). The proximal esophagus, the dorsal surface of the pharyngeal fold and the tonsil all contain simple, tubular mucus-secreting glands (*Sg*) which are invaded to varying degrees by lymphoid tissue. Recess (*R*) between the dorsal surface of the pharyngeal fold and proximal esophagus, tonsillar crypts (*C*) and branches of the crypts (*Cb*). **c**: Longitudinal section as indicated by the dotted line on the inset. Note the numerous crypts which open via fossules (*dotted arrows*) into the shallow tonsillar sinus (*Ts*) (*double-headed arrow*) before opening into the oropharynx. The pharyngeal tonsil is separated from the pharyngeal fold by a loose connective tissue capsule (*Lct*). Simple, branched, tubular mucus-secreting glands (*Gl*), ventral (*Pfv*) and dorsal (*Pfd*) surfaces of the pharyngeal fold, lymph nodules (*) and inter-nodular (*Ilt*) lymphoid tissue, connective tissue (*Ct*) and proximal esophagus (*Pe*).

The gross morphology of the pharyngeal folds of *D. novaehollandiae *[11] and *S. camelus *[12-14] have been described. In *D. novaehollandiae* a smooth, rounded, caudo-lateral extension of the fold originated from the dorsal surface of each fold (Figures 1a, 2a, 3) and enclosed a blind-ending recess (tonsillar crypt) between itself and the dorsum of the pharyngeal fold [11] (Figures 3a, b, c, 4a, b). The base of the caudo-lateral extension was continuous with the proximal esophagus (Figure 2a). This caudo-lateral extension denoted and formed part of the pharyngeal tonsil in *D. novaehollandiae* (see results below). In *S. camelus*, on the caudo-lateral surface of each pharyngeal fold, and situated retropharyngeally (dorsal to the esophageal mucosa), was an oval, raised structure which ran obliquely across the dorsal aspect of the attached part of the pharyngeal folds (Figures 1b, 2b, 5a). This oval structure opened to the oropharynx on the caudo-lateral edge of each fold (Figures 5a, b) and was positioned between the pharyngeal fold and proximal esophagus (Figure 5b). This structure represented the pharyngeal tonsil in *S. camelus* (see results below). Numerous longitudinally oriented crypts were present (Figures 5b, c, 6). The entrance to the tonsil displayed a shallow tonsillar sinus into which a number of tonsillar fossules opened (Figures 5b, 6c) (see below). Viewed from the oropharynx, the tonsillar sinus displayed a set of vertically oriented parallel vanes (Figure 5b). These vanes formed the walls of the crypts.

### Histology

**Figure 7:**
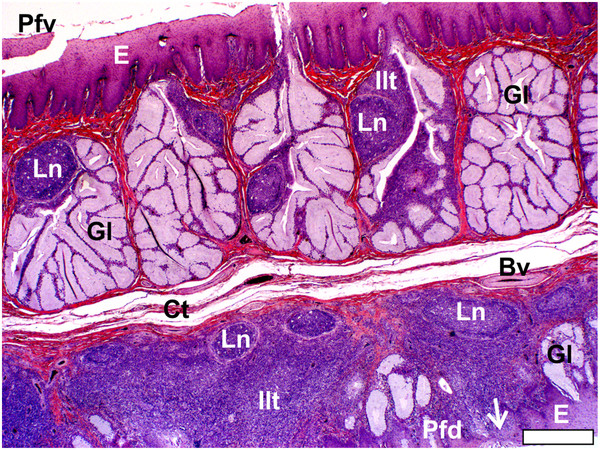
**Pharyngeal fold and non-follicular tonsil of *S. camelus.*** The ventral (*Pfv*) and dorsal (*Pfd*) surfaces of the pharyngeal fold are lined by a non-pigmented, non-keratinised stratified squamous epithelium (*E*) and separated by a layer of loose connective tissue (*Ct*). The lymph nodules (*Ln*) and inter-nodular lymphoid tissue (*Ilt*) are associated with simple, branched, tubular mucus-secreting glands (*Gl*) in the ventral surface of the fold and constitute *Lymphnoduli pharyngeales*. In the dorsal surface the lymphoid tissue is situated in the *Lamina propria* directly below the epithelium (non-follicular tonsil) which in places is very thin (*arrow*). Simple, tubular mucus-secreting glands and occasional simple, branched, tubular glands are present on the dorsal surface. Blood vessel (*Bv*). Bar = 400 μm.

The pharyngeal folds in both *D. novaehollandiae* and *S. camelus* displayed two surfaces; a ventral surface facing the oropharyngeal cavity (Figure 1) and a dorsal surface (Figure 2), forming the ventral boundary of the recess between this surface of the pharyngeal folds and the proximal esophagus (Figures 3d, 4a, 6a). In both *D. novaehollandiae* and *S. camelus*, the ventral surface of the pharyngeal folds typically displayed large, simple, branched tubular mucus-secreting glands (Figures 4, 6, 7). In both species many of the glands were associated with variably sized aggregations of lymph nodules and inter-nodular lymphoid tissue (Figures 4, 6, 7) which represented the *Lymphonoduli pharyngeales*[18]. The surface of the pharyngeal folds (ventral and dorsal) was covered by a non-keratinised, non-pigmented, stratified squamous epithelium (Figure 7) which was thicker on the ventral surface. The dorsal surface of the pharyngeal folds, which effectively formed the floor of the recess (Figures 3d, 4a, 6a), in both species, displayed mainly simple tubular mucus-secreting glands with an occasional simple, branched tubular gland being observed near the periphery (Figure 7). These glands were obliterated in places by dense aggregations of lymph nodules an inter-nodular lymphoid tissue (Figure 7). The lymphoid tissue was present in varying amounts, was not associated with the glands but situated in the *Lamina propria* directly below the surface epithelium (Figures 4, 6, 7) and represented a small non-follicular pharyngeal tonsil [3]. The overlying epithelium was either intact or invaded by the lymphocytes. The ventral and dorsal surfaces of the folds were separated from each other by a layer of relatively loose connective tissue in *S. camelus* (Figures 6, 7) and by dense irregular connective tissue in *D. novaehollandiae* (Figure 4).

In *D. novaehollandiae*, that portion of the dorsal surface of the pharyngeal fold enclosed by the caudo-lateral extension, together with the ventral surface of the extension, displayed a considerable amount of lymphoid tissue (Figure 3) consisting of lymph nodules, inter-nodular lymphoid tissue, mucus glands and intervening connective tissue septa (Figure 4). Enclosed between the two lymphoid tissue surfaces was a central crypt which opened into the oropharynx (Figures 2a, 3d, 4a, b). Only a single central crypt was obvious which displayed various degrees of branching (Figure 4). This tissue represented a follicular pharyngeal tonsil [19] in *D. novaehollandiae* and was surrounded by an ill-defined capsule. The part of the caudo-lateral extension protruding beyond the pharyngeal fold displayed simple, branched, tubular mucus-secreting glands on its ventral surface, interspersed with varying amounts of lymph nodules and inter-nodular lymphoid tissue. Its dorsal surface was morphologically similar to that of the surrounding pharyngeal fold, displaying simple tubular mucus-secreting glands and varying amounts of lymph nodules and inter-nodular lymphoid tissue (Figure 4). The two surfaces of the caudo-lateral extension were separated by a core of loose connective tissue (Figure 4).

**Figure 8:**
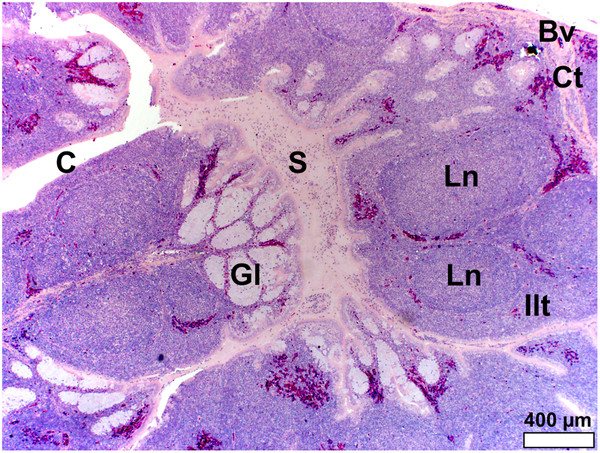
**Follicular pharyngeal tonsil of *S. camelus.*** Note the simple, tubular, mucus-secreting glands (*Gl*) which are invaded by lymphoid tissue, forming lymph nodules (*Ln*) and inter-nodular lymphoid tissue (*Ilt*). Glandular secretion (*S*) fills the tonsillar crypts (*C*). Connective tissue septa (*Ct*) surround the lymphoid tissue and distribute larger blood vessels (*Bv*).

**Figure 9:**
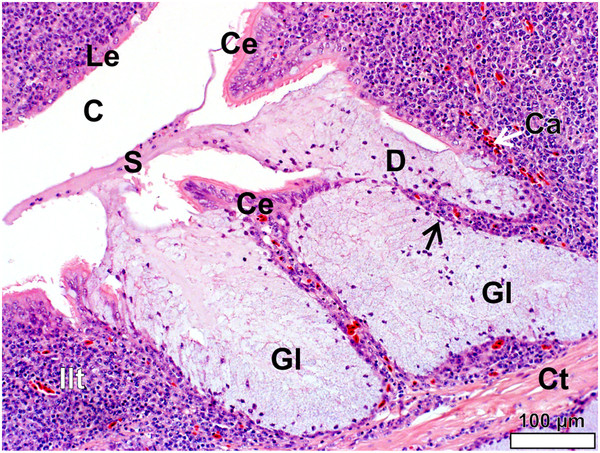
**Simple, tubular mucus-secreting glands (Gl) in the follicular pharyngeal tonsil of *S. camelus.*** The secretion (*S*) appears to be formed by the release of whole mucus cells from the glandular epithelium. Both a round/cuboidal lymphoepithelium (*Le*) and ciliated columnar lymphoepithelium (*Ce*) line the tonsillar crypt (*C*). Connective tissue (*Ct*), inter-nodular lymphoid tissue (*Ilt*), capillaries (*Ca*), gland duct (*D*) and basally compressed nucleus of mucus cell (*black arrow*).

In *S. camelus* the follicular pharyngeal tonsil present on the dorsal surface, although located retropharyngeally, was situated in a similar region on the pharyngeal folds to that in *D. novaehollandiae* and was encapsulated by loose connective tissue (Figure 6). The lymphoid tissue consisted of lymph nodules and inter-nodular lymphoid tissue surrounding a central crypt, which displayed varying degrees of branching (Figure 6). This branching was more pronounced in *S. camelus* than in *D. novaehollandiae*. However, in contrast to the situation in *D. novaehollandiae*, numerous crypts opened into the oropharynx in *S. camelus* (Figures 5b, 6c). The tonsillar parenchyma was divided by numerous connective tissue septa (Figures 5c, 6). Mucus-secreting glands (presumably simple tubular) were present (Figures 8, 9). However, it was difficult to appreciate their structure as they were often obliterated by invading lymphoid tissue (Figure 6). Unlike in *D. novaehollandiae*, some of the crypts were filled with mucus and cell debris (Figure 8).

**Figure 10:**
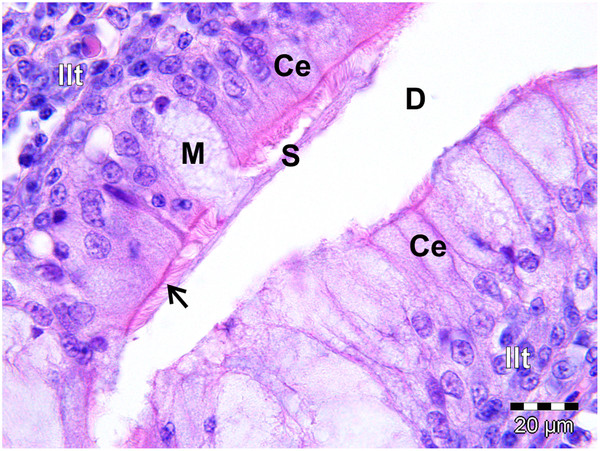
**Ciliated columnar lymphoepithelium (Ce) lining a gland in the follicular pharyngeal tonsil of *S. camelus.*** The mucus-secreting gland has been invaded by lymphocytes of the inter-nodular lymphoid tissue (*Ilt*). The cilia (*arrow*) spread the secreted mucus (*S*) from the mucus cell (*M*) into the gland duct (*D*).

**Figure 11:**
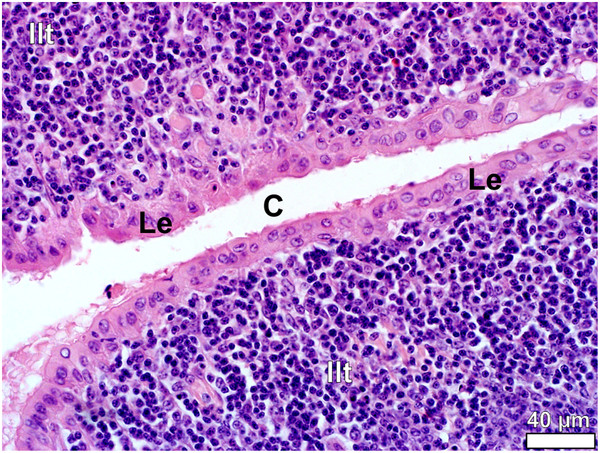
**Lymphoepithelium (Le) in the follicular pharyngeal tonsil of *D. novaehollandiae.*** The tonsillar crypt (*C*) is lined by typical round/cuboidal lymphoepithelium consisting of a single to double layer of rounded cells. The lymphoepithelium overlies inter-nodular lymphoid tissue (*Ilt*).

**Figure 12:**
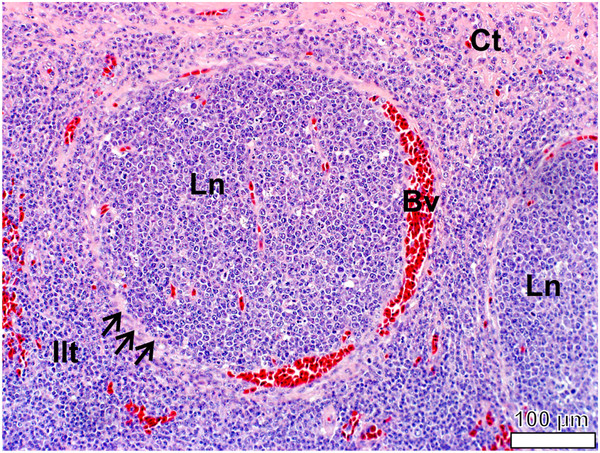
**Follicular pharyngeal tonsil of *S. camelus.*** The lymph nodules (*Ln*) are surrounded by a ring of delicate connective tissue (*black arrows*) richly supplied with blood vessels (*Bv*). Inter-nodular lymphoid tissue (*Ilt*) is situated between the lymph nodules and through which course prominent connective tissue (*Ct*) septa.

**Figure 13:**
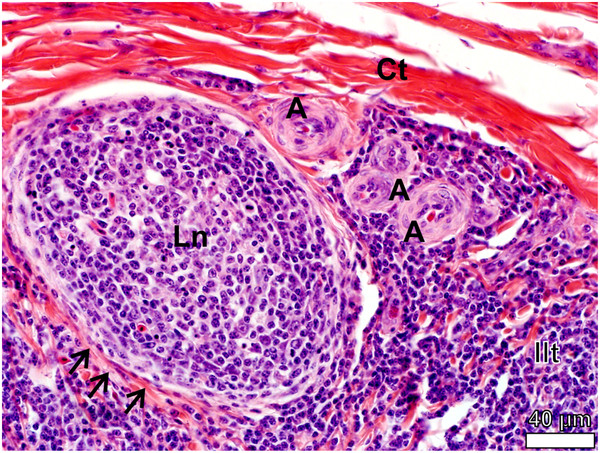
**Lymph nodule (Ln) in the follicular pharyngeal tonsil of *D. novaehollandiae.*** The lymph nodule is surrounded by delicate connective tissue (*black arrows*) and a series of arterioles (*A*) are present in the inter-nodular lymphoid tissue (*Ilt*). Connective tissue (*Ct*).

**Figure 14:**
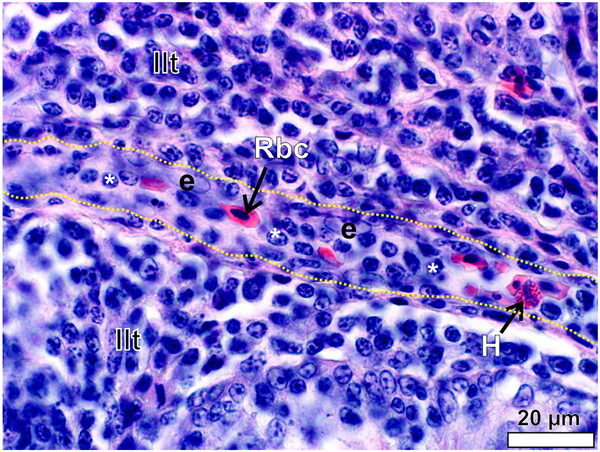
**A venule traversing the inter-nodular lymphoid tissue (Ilt) in the follicular pharyngeal tonsil of *S. camelus.*** The wall of the venule is roughly outlined (*yellow dotted lines*) for clarity. The endothelial nuclei (*e*) are pale and oval to round. Note the high number of lymphocytes (*) within the lumen. Red blood cell (*Rbc*) and heterophil (*H*).

In both species the epithelium lining the crypts varied in structure from a stratified squamous epithelium to a psuedostratified ciliated columnar epithelium (associated with the glands) (Figures 9, 10) or a single to double (Figures 9, 11) layer of round/cuboidal cells with round/oval nuclei. In places the epithelium appeared absent, its position being occupied by concentrations of lymphoid tissue. Lymphocytes were observed to traverse both the psuedostratified ciliated columnar epithelium (Figures 9, 10) and the epithelium composed of round/cuboidal cells (Figures 9, 11). In places this took the form of individual or small groups of lymphocytes distorting the base of the epithelial lining cells and forming small pockets in which they were housed. These features were typical of specialised epithelial cells, the M cells, the presence of which, together with the infiltrating lymphocytes, is indicative of a lymphoepithelium [1,2] or follicle-associated epithelium [1]. The lymphoid tissue was well vascularised (Figures 9, 10, 11, 12, 13, 14) and the capillaries and venules were lined by endothelial cells with round to oval, pale nuclei (Figure 14). Many lymphocytes were present in the lumen of the blood vessels (Figure 14), showing a high degree of trafficking. Lymph nodules were surrounded by a delicate layer of connective tissue (Figures 12, 13).

## Discussion

The existence of tonsils in the oropharynx of ratite species was first reported by Cho et al. [15] who noted that “The ostrich tonsils and tongue are smooth, blunt and U-shaped. In the Darwin's rhea both tongue and tonsils have simple, pointed V-shaped tips. The tonsils in the emu are similar to the rhea but have a small flap laterally”. However, the term ‘tonsil’ appears to have been used inappropriately and it seems more probable from the foregoing description [11], that what is referred to as ‘tonsils’ [15] are in fact the pharyngeal folds. The relatively large amount of lymphoid tissue present in the pharyngeal folds of *D. novaehollandiae *[16] and *S. camelus*[14] has also prompted more recent reference to these structures as tonsils. The question therefore arises as to whether the pharyngeal folds in *D. novaehollandiae* and *S. camelus* represent true pharyngeal tonsils? Tadjalli et al. [13] briefly mention the presence of pharyngeal tonsils in *S. camelus,* describing them as “…two pockets like diverticuli that are bordered by a prominent circular pharyngeal fold. Each pocket has an oval structure, called pharyngeal tonsil” [sic]. Although the opening to the tonsil and its oval shape were depicted and the structures correctly identified as tonsils, no macro- or microscopic evidence was provided to support this conclusion. A pharyngeal tonsil has recently been reported in *D. novaehollandiae* but the distinction between this structure and the pharyngeal folds is still unclear [20].

The present study provides definitive morphological evidence that the pharyngeal folds *per se* do not represent tonsils (as defined in mammals), but rather lymphoid tissue-rich regions which are closely associated with true pharyngeal tonsils. Much of the confusion surrounding the naming of “tonsils” in birds stems from the fact that the GALT, in the form of aggregated lymph nodules, observed in the oropharynx of a number of bird species [4-7,9], has traditionally been interpreted as constituting pharyngeal tonsils.

GALT occurs in two forms, namely, (1) as solitary and aggregated lymph nodules and inter-nodular lymphoid tissue assembled below the surface epithelium, and (2) as tonsils [21]. Two types of tonsils are recognised, those with follicles, and those without [1,3]. A follicular tonsil comprises one or more tonsillar follicles of which each follicle is “composed of a crypt, its orifice (fossula), and its surrounding lymphatic tissue, which contains Lymphonoduli” [19]. Non-follicular tonsils bulge into the oropharynx and have a slightly folded epithelium [22], are devoid of crypts but contain lymph nodules and inter-nodular lymphoid tissue, an example being the tonsil of the soft palate and tubal tonsil of the ruminant [3]. According to *Nomina Anatomica Veterinaria* (NAV) [19] the term “tonsil” (*Tonsilla*) is applied to GALT present in the pharynx. Immunologically active lymphoid tissue in other parts of the digestive tract not associated with crypts or fossula is referred to as *Lymphonoduli solitarii* (solitary lymph nodules) and *Lymphonoduli aggregati* (aggregated lymph nodules) in the small and large intestine and as *Lymphonoduli gastrici*, which are associated with the glands in the stomach [19]. Similarly, the aggregated lymph nodules of GALT found in specific anatomical locations in birds have been grouped under the term *Lymphonoduli aggregati apparatus digestorii* according to *Nomina Anatomica Avium*[8]. These include the *Lymphonoduli pharyngeales*, *Lymphonoduli esophageales*, *Lymphonoduli cecales* and *Lymphonoduli rectales*[8]. However, the term *Lymphonoduli* is deemed, in avians at least, to be synonymous with tonsils and these tonsils are defined as “Lymphonoduli aggregati of relatively constant occurrence and relatively large size” [8]. The definition of an avian tonsil thus clearly differs from that of a mammalian tonsil. Further complicating this anomalous situation is the occurrence in *Gallus domesticus* of tonsils conforming to the classical description of the mammalian tonsil (except that they are not located in the pharynx). These include esophageal [10,23], cecal [24] and pyloric [21] tonsils. Thus in the avian literature the distinction between *Lymphonoduli* (*solitarii* and *aggregati*) and *Tonsilla* has become blurred, leading to the term tonsil being used synonymously for both forms of GALT in birds. The fact that a typical “mammalian” tonsil has not previously been described in the avian oropharynx has also contributed to the inappropriate use of nomenclature.

The identification of a follicular pharyngeal tonsil (*Tonsilla pharyngea*) (as defined by NAV [19]) as a component of GALT in the oropharynx of *D. novaehollandiae* and *S. camelus* warrants stricter and more consistent use of the terms “aggregated lymph nodules (*Lymphonoduli aggregati*)” and “tonsils (*Tonsilla*)” in birds. Thus to avoid confusion these terms should no longer be considered synonymous and the GALT present in the pharyngeal region (including the pharyngeal folds) of birds not forming typical tonsils (follicular or non-follicular), should be named pharyngeal lymph nodules (*Lymphonoduli pharyngeales*). However, it should be noted that the large aggregations of lymph nodules and inter-nodular lymphoid tissue situated directly below the pharyngeal epithelium on the dorsal surface of the pharyngeal folds in *D. novaehollandiae* and *S. camelus*, also represents a small pharyngeal tonsil. The lack of tonsillar crypts in this concentration of lymphoid tissue defines this tonsil as a non-follicular pharyngeal tonsil. True tonsils which display similar morphological features to those described in *D. novaehollandiae* and *S. camelus* have been described in *Gallus domesticus*. However, these tonsils are not located in the pharyngeal region and are positioned in other parts of the digestive tract such as the cecum [24], esophagus [10] and pylorus [21].

The pharyngeal folds of both *D. novaehollandiae*[11] and *S. camelus *[14] share similar features. In contrast, the pharyngeal folds of *Rhea americana* are much reduced and display no free portion [25]. However, on the caudo-lateral edge of each pharyngeal fold in this species is an oval opening which leads to a small pocket in the fold (personal observation). This opening and pocket in *R. americana* is similar in its location to the pharyngeal tonsil and its opening in *S. camelus* and is also situated retropharyngeally (personal observation). It is therefore highly suggestive that if *D. novaehollandiae* and *S. camelus* display pharyngeal tonsils in a similar location to each other, and that if *R. americana* possesses a similar structure in a similar region of the pharyngeal fold to that in *S. camelus*, that it too possesses a pharyngeal tonsil. The three ratite species mentioned above each belong to a different order (*Casuariiformes*, *Struthioniformes* and *Rheiformes*) and it would appear that pharyngeal tonsils are unique to *D. novaehollandiae*, *S. camelus* and possibly also to *R. americana*. It would be of great interest to investigate whether the remaining orders (*Apterygiformes* and *Tinamiformes*), as well as *Casuarius* spp. from the same order as *D. novaehollandiae*, also display pharyngeal tonsils, which could make these structures a characteristic feature of the Superorder *Paleognathae*.

### Function

The pharyngeal folds in *D. novaehollandiae* and *S. camelus* fulfil mechanical functions (respiratory and digestive) and an immunological function. The respiratory function has been addressed [11] in *D. novaehollandiae*. The digestive function of the folds is reflected by the presence of many large, simple, branched tubular mucus-secreting glands (*Gl. tonsilla pharyngea*) in *D. novaehollandiae* which provide lubrication for food boli and also protect the non-keratinised epithelium [16]. The immunological function is expressed by the significant concentration of GALT in the form of aggregated lymph nodules (*Lymphonoduli pharyngeales* and the non-follicular tonsil) in the pharyngeal folds, and by the massive accumulation of GALT in the closely associated follicular tonsil. Thus, the pharyngeal folds in *D. novaehollandiae* and *S. camelus* represent the first strategic, anatomical location of GALT in these species. The placement of the pharyngeal tonsils at this specific location appears to be related to the catch and throw or cranio-inertial feeding method employed by ratite species [26,27] which results in the food items travelling from the bill-tip to land in the proximal esophagus prior to being swallowed. Thus, the first point of contact of the food (carrying potentially harmful antigens) with a mucosal surface is that of the pharyngeal folds [16] and the associated follicular pharyngeal tonsils.

In mammals, tonsils do not contain salivary glands [28] but are, however, surrounded by salivary glands. The secretion from these glands washes out accumulated leukocytes along with other microorganisms from the tonsillar crypts and fossules to prevent infection [28]. In contrast to mammals, the pharyngeal tonsils of *D. novaehollandiae* and *S. camelus* contain varying amounts of salivary glands in the form of simple, tubular mucus-secreting glands. The mucus glands in the tonsils of *D. novaehollandiae* and *S. camelus* may similarly function to prevent the accumulation of leukocytes and microorganisms in the tonsillar crypts, thus preventing infection. In mammals this is achieved by secretions from the surrounding salivary glands washing out debris [28] and by the action of swallowing which compresses the crypts. In birds, which lack an upper esophageal sphincter, the compression force of swallowing is largely absent and the primary method of cleaning the tonsillar crypts would depend on the pressure exerted from the build-up of secreted mucus.

The follicular pharyngeal tonsils of *D. novaehollandiae* and *S. camelus* contain numerous tonsillar crypts and these invaginations reportedly result in a higher concentration of lymphoid tissue in a particular location [21,28]. This fact points to the importance and immunological significance of the pharyngeal tonsils in *D. novaehollandiae* and *S. camelus*, as in common with other avian species, both *D. novaehollandiae* and *S. camelus* display significant accumulations of lymphoid tissue (GALT) in the oropharynx, but not necessarily forming pharyngeal tonsils. Thus, in addition to the normal component of GALT (*Lymphonoduli pharyngeales*) found in the avian oropharynx, *D. novaehollandiae* and *S. camelus* possess follicular tonsils, thus massively increasing the volume of lymphoid tissue present in the oropharynx. Therefore, the oropharynx of *D. novaehollandiae* and *S. camelus* is immunologically highly protected, in contrast to the situation in other described avian species.

## Conclusions

The pharyngeal region of the oropharynx of *D. novaehollandiae* and *S. camelus* demonstrates a high density of lymphoid tissue. Based on the location and morphological features of this tissue three specific immunological entities can be distinguished, namely, a prominent, well-defined follicular pharyngeal tonsil; a small yet defined non-follicular tonsil; and an extensive field of aggregated lymph nodules (associated with the glands) confined largely to the ventral surface of the pharyngeal folds. The follicular pharyngeal tonsil in both species described in this study demonstrate the typical morphological features that characterise and define the pharyngeal tonsil in mammals [3,10,19,28], and differentiate these two ratite species from other known avians where similar structures have not been described. The pharyngeal tonsil, especially in *S. camelus*, represents a well-defined organ, and differs markedly from the lymphoid tissue (*Lymphonoduli pharyngeales*) located in the pharyngeal region of other avian species in which this accumulation of GALT has been termed the pharyngeal tonsil. The true pharyngeal tonsil of *D. novaehollandiae* and *S. camelus* is a follicular tonsil, which in the latter species is polycryptic and displays a shallow tonsillar sinus.

Lymphoepithelium lines parts of the crypts in the pharyngeal tonsils of *D. novaehollandiae* and *S. camelus*. This specialised epithelium (also termed follicle-associated epithelium [1]), in *G. domesticus*, performs an important role in the sampling of antigens [29]. As MALT is not supplied by afferent lymphatics, the presence of specialised M cells allows for the uptake of antigens [2,30]. The presence of lymph nodules, inter-nodular lymphoid tissue and lymphoepithelium in the GALT in the pharyngeal folds and tonsils would suggest these structures to be inductive sites [1]. At these sites IgA class switching and clonal expansion of B-cells would occur in response to antigen specific T-cell activation, following which, the activated B- and T-cells would migrate to effector sites [1]. M cells have a distinct ultrastructural appearance but are difficult to distinguish by light microscopy and clusters of lymphocytes within the epithelium may be the only indication of the presence of these cells [1]. The lymphoepithelium, together with the putative M cells, present in the pharyngeal folds and tonsils of *D. novaehollandiae* and *S. camelus*, represent a significant area of immunological surveillance, as was suggested for the apical caecal diverticulum of *G. domesticus*, which also displays lymphoid tissue lined by follicle-associated epithelium [31]. The pharyngeal tonsils in *D. novaehollandiae* and *S. camelus* may pose a suitable and easily accessible site for immune response surveillance as indicated by swelling and inflammation of the tonsillar tissue and pharyngeal folds. This would be facilitated by the fact that the heads of these commercially slaughtered ratites are discarded, thus sampling at these sites would not result in financial losses. Further studies will be needed to clarify the full immunological significance of the pharyngeal lymph nodules, the non-follicular pharyngeal tonsil and the follicular pharyngeal tonsil in *D. novaehollandiae* and *S. camelus*.

## Methods

We collected the heads of 8 sub-adult (14–15 months) *D. novaehollandiae* and 8 sub-adult (12–14 months) *S. camelus* of either sex from accredited commercial abattoirs (Oryx Abattoir, Krugersdorp, Gauteng Province, South Africa [*D. novaehollandiae*] and the Klein Karoo Ostrich abattoir, Oudtshoorn, Western Cape Province, South Africa [*S. camelus*]) immediately after slaughter of the birds. The heads were rinsed in running tap water to remove traces of blood and mucus and then immersed in plastic buckets containing 10% neutral-buffered formaldehyde. The beaks were propped open using a small wooden block to ensure adequate fixation of the oropharynx.

The heads were opened (Figure 1) as previously reported [11] to reveal the roof of the oropharynx and the relevant anatomical structures described. The pharyngeal folds from both species were excised by making sharp incisions just below the level of the choana, laterally, and by incising the proximal esophagus and freeing the folds and proximal esophagus from the connective tissue of the retropharyngeal region. The pharyngeal tonsil in *S. camelus* was exposed by removal of the tissues attaching the pharyngeal folds and adjacent structures to the retropharyngeal region (Figures 2b, 5a). Macroscopic features were photographed using a Canon EOS 5D digital camera (Canon, Japan) with a 28–135 mm lens and a Nokia N8 equipped with a 12 megapixel camera and a Zeiss lens. One pharyngeal tonsil was injected with Indian ink (via the tonsillar sinus) in *S. camelus* to stain and demonstrate the crypts and connective tissue septa (Figure 5) and then serially sectioned in the transverse plane. A pharyngeal tonsil from *D. novaehollandiae* was similarly sectioned (Figure 3) to demonstrate its macroscopic structure. Transverse sections were viewed and micrographed using an Olympus SZX16 stereomicroscope (Olympus Corporation, Tokyo, Japan) equipped with a DP72 camera and Olympus cellSens imaging software (Olympus Corporation, Tokyo, Japan).

For light microscopy, the pharyngeal folds and adjoining tissues were removed (as described above) from 5* D. novaehollandiae* and 5 *S. camelus* heads. The tissue samples were cut into appropriate longitudinal and transverse sections and routinely prepared for light microscopy using a Shandon model 2LE Automatic Tissue Processor (Shandon, Pittsburgh, PA, USA). Sections were stained with Haematoxylin and Eosin (H&E) and viewed and micrographed using an Olympus BX63 light microscope (Olympus Corporation, Tokyo, Japan) equipped with a DP72 camera and Olympus cellSens imaging software.

The terminology used in this study was that of Nomina Anatomica Avium [18] and Nomina Anatomica Veterinaria [19]. This work was approved by the Animal Use and Care Committee of the Faculty of Veterinary Science, University of Pretoria.

## Competing interests

The authors declare that they have no competing interests.

## Authors' contributions

MRC took the primary lead in the compilation of the manuscript. The concept was the original idea of MRC and elaborated upon by JTS. JTS acted in a supervisory role on all aspects of the work and was responsible for the refinement of the manuscript. Both authors collected the specimens, discussed the results and contributed equally to the manuscript.

## Authors' details

MRC is a Senior Lecturer and PhD student in the Department of Anatomy and Physiology, Faculty of Veterinary Science, University of Pretoria. MRC's research focus is on the detailed comparative anatomy of the oropharynx of ratite species. JTS is a Professor in the same department as MRC and has 25 years of experience working on various aspects of ratite anatomy. The research of MRC and JTS comprises detailed descriptive studies aimed at elucidating underlying functions.
